# A rare interstitial lung disease in young adulthood due to surfactant protein C gene mutation: Two case reports with brief literature review

**DOI:** 10.1016/j.radcr.2025.05.051

**Published:** 2025-06-12

**Authors:** Yildiz Sengul, Emily Gosche, Todd Hazelton

**Affiliations:** a Department of Radiology, University of North Carolina, 101 Manning Dr, Chapel Hill, NC 27514; b Division of Pulmonary Disease and Critical Care Medicine, University of North Carolina, 101 Manning Dr, Chapel Hill, NC 27514

**Keywords:** Surfactant Protein C, SP-C gene mutation, Adulthood interstitial lung disease, Pulmonary fibrosis, CT scan

## Abstract

Interstitial lung disease associated with mutations in the surfactant protein C gene (SFTPC) is a rare condition. These mutations can be inherited as an autosomal dominant trait or occur sporadically due to a de novo mutation. The clinical symptoms of this disease can vary widely, ranging from fatal acute respiratory distress syndrome (RDS) in neonates to chronic lung disease in adults. We present 2 cases of young adults with SFTPC mutations related to interstitial lung disease (ILD). Chest CT findings in these cases included ground-glass opacities, reticulation, multiple cysts, and thickening of the interlobular septae.

## Introduction

Pulmonary surfactant is a complex mixture of lipids and proteins that lower the surface tension at the air-liquid interface, preventing end-expiratory atelectasis. Surfactant contains approximately 90% lipid and 10% protein by weight, including specific proteins whose expression is highly enriched in lung tissue [1]. Surfactant Protein C (SP-C), produced in alveolar type II cells, is a small hydrophobic protein that stabilizes and enhances the spreading of surfactant phospholipids along the alveolar surface. This process reduces surface tension within the alveoli, prevents alveolar collapse during expiration, and maintains lung compliance. In addition to its biophysical roles, SP-C is important in regulating inflammation and cell differentiation [2].

Mutations in the surfactant protein C gene (SFTPC) lead to misfolding of the large proSP-C and subsequent accumulation in type II alveolar cells. This abnormality activates a pro-inflammatory cascade that triggers cellular death and recruitment of T-cells and fibroblasts, eventually leading to interstitial lung disease (ILD) [3]. Mutations in the SFTPC gene are inherited as an autosomal dominant trait with variable penetrance or they can arise sporadically from a de novo mutation [4]. SP-C deficiency caused by SFTPC mutations can exhibit a variety of clinical symptoms, ranging from severe respiratory distress in infants to chronic interstitial lung disease in adults. Recognizing these varying manifestations is crucial for early intervention and effective management [5].

Chest computed tomography (CT) findings are essential for diagnosing and assessing the extent of lung involvement in SP-C deficiency. These findings include ground-glass opacities, reticulation, cysts, and thickening of interlobular septae. Ground glass opacities typically decrease with age, while lung cysts generally tend to increase. Ground-glass opacities on CT are associated with diffuse alveolar septal thickening, type II alveolar cell hyperplasia, and accumulation of macrophages within the alveoli. In contrast, lung cysts are associated with the dilation of the respiratory bronchioles and alveolar ducts [6]. This case series aimed to investigate surfactant protein C deficiency as a potential diagnosis in young adults with CT findings suggestive of interstitial lung disease and to recommend genetic testing.

## Case presentation

### Case 1

A 27-year-old male presented with history of progressive dyspnea, exercise intolerance, chest pain, recurrent pneumothoraxes and chronic cough. He was first known to have pulmonary disease in early childhood, but was lost to follow-up. His family history includes several relatives with lung disease who died at young ages. His maternal grandmother died from an unspecified lung disease in her 30s. His sister needed a lung transplant at the age of 7 and unfortunately passed away during her teenage years. The pathological diagnosis for her lung condition was desquamative interstitial pneumonia (DIP).

**Fig. 1:**
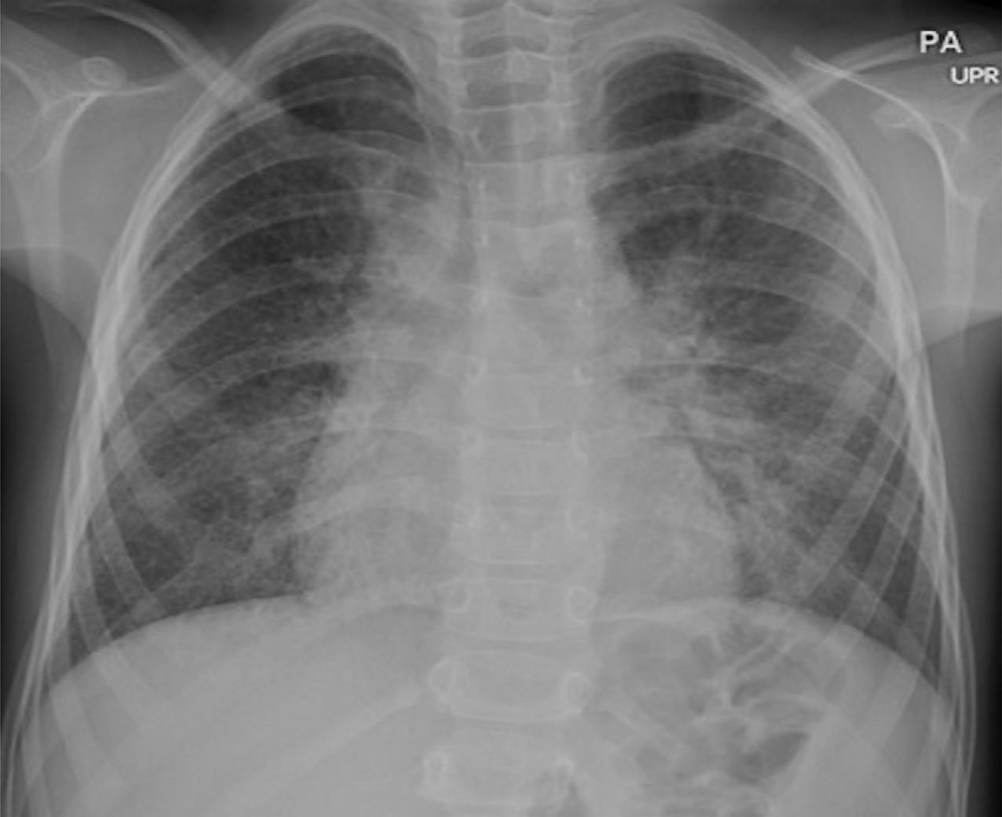
At 5 years of age (case 1), PA chest radiography shows peribronchovascular thickening with bilateral diffuse interstitial thickening.

**Fig. 2:**
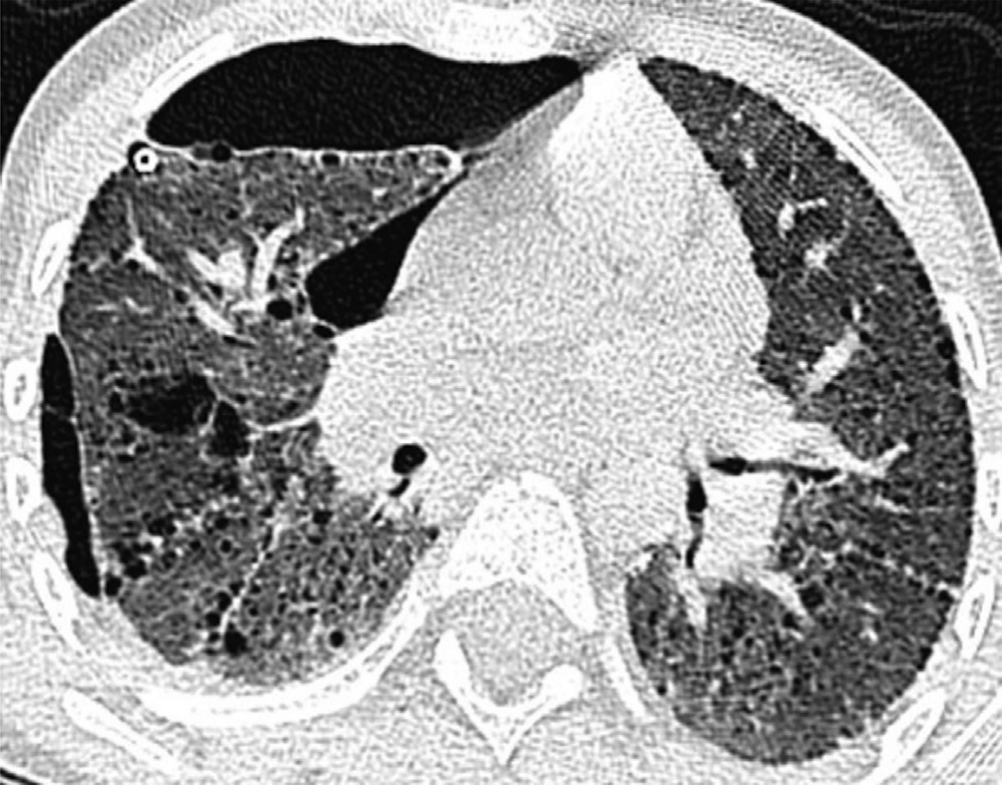
At 7 years of age (case 1), an axial noncontrast chest CT slice shows multiple thin-walled cysts of varying sizes, predominantly distributed in subpleural, juxtafissural, and peribronchovascular regions of both lungs, as well as a right-sided pneumothorax.

**Fig. 3:**
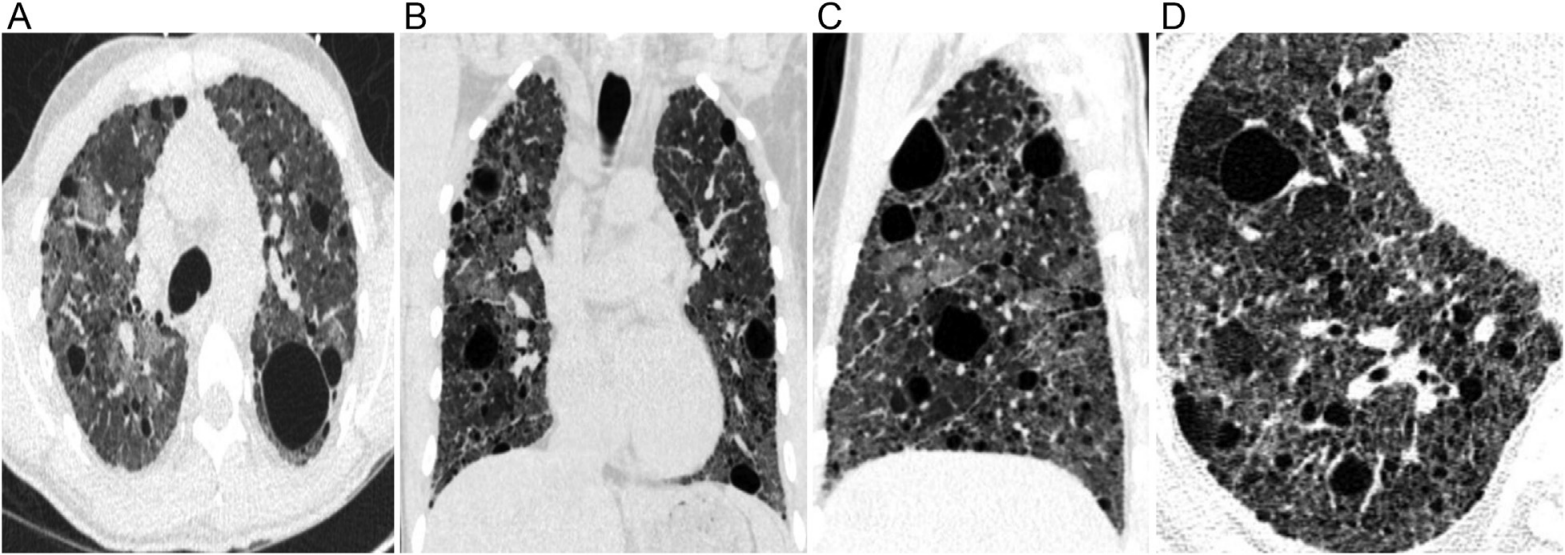
Imaging findings at 27 years of age (case 1). Axial (A) and coronal (B) noncontrast-enhanced chest CT slices demonstrate patchy ground glass opacities, reticulations, interlobular septal thickening, and multiple variable size thin-walled cysts throughout the lungs. A sagittal image through the right lung (C) demonstrates the diffuse distribution of the findings. Small field of view axial image (D) demonstrates subpleural and central reticulations with thickening of bronchovascular bundles consistent with pulmonary fibrosis.

Pulmonary function tests (PFT) revealed a restrictive lung disease pattern characterized by a reduced lung volume with a total lung capacity (TLC) that was 41% predicted as well as impaired gas exchange and diffusion capacity of the lungs (DLCO) that was severely reduced at 15% predicted. A transthoracic echocardiogram indicated moderate dilation of the right ventricle and mild dilation of the right atrium. A right heart catheterization revealed mild pulmonary hypertension with a mean pulmonary artery (mPA) pressure of 32 mmHg, pulmonary vascular resistance 4.22 and a postcapillary wedge pressure (PCWP) of 13 mmHg. A chest radiography at 5 years of age demonstrated diffuse increased interstitial density in both lungs (Fig. 1). A chest CT scan at age 7 revealed multiple thin-walled cysts of variable sizes throughout the subpleural, peribronchovascular, and perifissural areas of the lungs, as well as a right pneumothorax (Fig. 2). A follow-up CT scan performed at age 27, indicated the presence of patchy ground-glass opacities, multiple subpleural and intraparenchymal diffuse cysts, as well as reticulations, thickening of the interlobular septa (Fig. 3). With advancing age, there was progressive thickening and fibrosis of the interstitium. The imaging features indicate chronic cystic interstitial lung disease.

Genetic testing revealed a heterozygous mutation (c.314A>G) in the SFTPC gene, indicating an autosomal dominant SP-C deficiency.

### Case 2

A 32-year-old female presented with a significant decline in her respiratory status, experiencing dyspnea with minimal exertion. She was a healthy full-term infant, but at 7 months old, she began to develop respiratory issues characterized by difficulty breathing and failure to thrive. An open lung biopsy revealed the presence of red blood cells and hemosiderin-laden macrophages in the alveolar space, along with some foamy macrophages and sloughed type II pneumocytes. Additionally, there was mild interstitial thickening accompanied by fibroblasts and histiocytes. Eventually, she was diagnosed with interstitial lung disease in infancy, which was believed to be caused by pulmonary hemosiderosis and reflux. After receiving immunosuppression therapy, she showed improvement and remained stable without medication for over 20 years. At 23 years of age, she again developed a dry cough and dyspnea during strenuous activities and was evaluated for chronic lung disease which had been attributed to chronic reflux. She reported that repeated pH probe assessments and endoscopies revealed no evidence of chronic reflux. Despite these negative evaluations, she continued to experience dyspnea that intermittently worsened with extreme exertion. A series of tests was performed to determine the final diagnosis.

PFTs were obtained and revealed a restrictive pattern with a forced vital capacity (FVC) that was 52% predicted with a normal FEV1/FVC ratio; the DLCO was severely reduced at 45% the predicted value, additionally concerning for restrictive lung disease. Bronchoscopy with bronchoalveolar lavage was performed and revealed bronchial cells showing chronic inflammation with eosinophils. No hemosiderin or malignant cells were found.

**Fig. 4:**
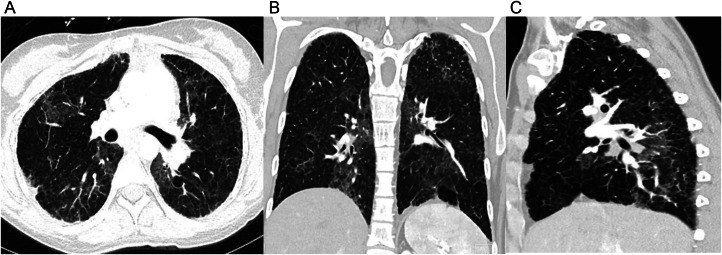
Imaging findings at 26 years of age (case 2). Axial (A), coronal (B), and sagittal (C) chest CT images show patchy ground glass opacities with interlobular septal thickening (crazy paving), small thin-walled cysts, and mild peripheral reticulations.

**Fig. 5:**
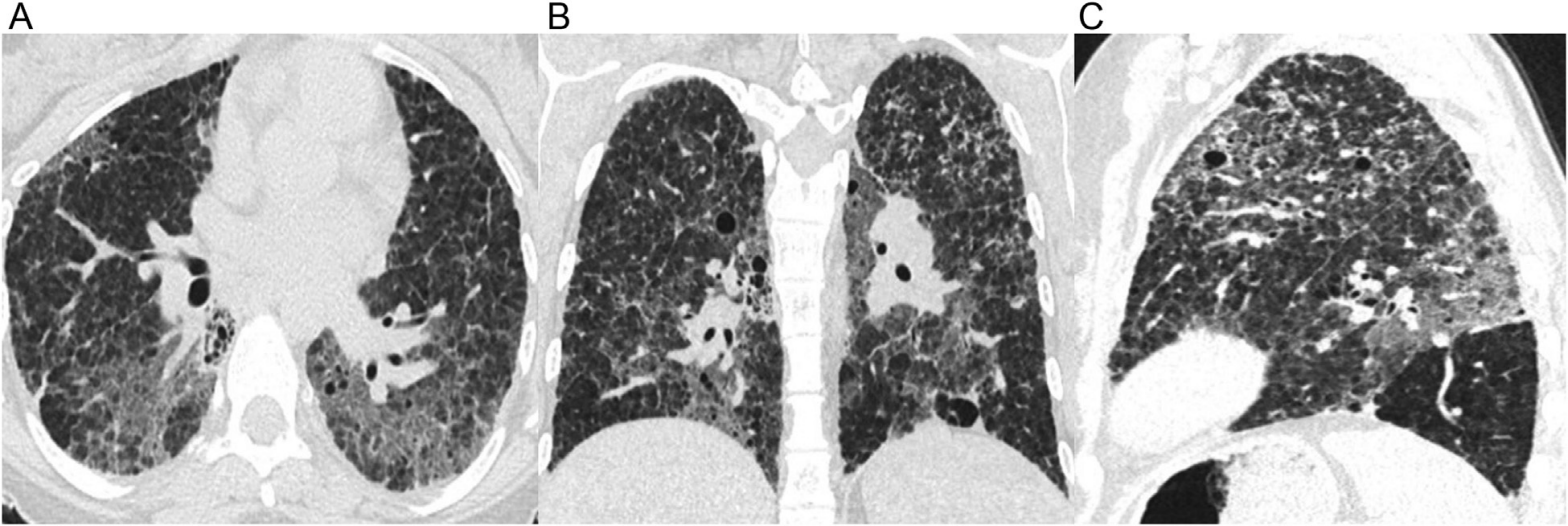
Imaging findings at 32 years of age (case 2). Axial (A), coronal (B), and sagittal (C) chest CT images display worsening findings, including patchy ground glass opacities with interlobular septal thickening, thin-walled cysts, and reticulations.

At age 26, a chest CT revealed scattered ground glass opacities with interlobular septal thickening and intralobular interstitial thickening in a “crazy paving” pattern of diffuse lung disease, as well as few small, thin-walled pulmonary cysts (Fig. 4). A follow-up CT scan performed when the patient was 32 years old showed an increase in the number of pulmonary cysts, worsened reticulations, and ground-glass opacities with interlobular septal thickening. (Fig. 5). The imaging findings indicated a progression of chronic cystic interstitial lung disease. Genetic testing revealed a heterozygous mutation in the SFTPC gene, the pathogenic variant (c.314A>G), indicating an autosomal dominant SP-C deficiency. Her father and mother underwent surfactant mutation genetic testing and were both negative for I73T variant.

## Discussion

SP-C is a hydrophobic protein synthesized in type II pneumocytes that plays an important role in stabilizing and facilitating the distribution of surfactant phospholipids within the alveolar space [1]. SFTPC mutations can be inherited in an autosomal dominant manner or can result from de novo mutations. Since their initial description by Noagee et al. in 2001, over 40 mutations in the SFTPC gene characterized by dominant expression have been identified [4,6,7]. While the exact pathophysiology of the lung disease remains unclear, it may be associated with the accumulation of misfolded proSP-C in type II alveolar cells, which leads to inflammation and cellular apoptosis. Destruction of type II alveolar cells hinders their ability to replenish type I alveolar cells following cellular injury, eventually resulting in pulmonary fibrosis [2].

SP-C deficiency is a rare lung disease with varying ages of onset, severity levels, and natural progression. Acute respiratory failure and interstitial lung disease are associated with this condition. The severity of symptoms can vary significantly, ranging from respiratory distress syndrome in neonates to adults who may remain asymptomatic for years. This variability is influenced by factors such as the specific type of mutation, modifier genes, age, and treatment options [4,[8], [9], [10], [11]].

Imaging findings on chest radiography typically show hazy pulmonary opacities and interstitial thickening that can be distributed in either a patchy or diffuse pattern. Chest CT, an essential modality for diagnosis and follow-up, most commonly demonstrates diffuse or patchy ground-glass opacities, reticulations, irregular thickening of the interlobular septae, and lung cysts. Rare findings on CT include bronchiectasis, pulmonary hypertension and diffuse alveolar hemorrhage. As patients age, there is a regression of ground-glass opacities with development of lung cysts, and increased septal thickening [6,[12], [13], [14]].

Limited literature available regarding the radiologic manifestations of surfactant protein C (SP-C) deficiency. Mechri et al. described the findings from thoracic CT scans in lung disease associated with SFTPC mutations. In their study, they found that the most common abnormalities were ground-glass opacities in 93% of cases and lung cysts in 40%. Other noted abnormalities included interlobular septal thickening (7%), consolidation (7%), and pulmonary nodules (7%)[6]. Tang et al. described 5 pediatric patients with surfactant protein C (SP-C) dysfunction in the first report linking SP-C dysfunction with diffuse alveolar hemorrhage in 2 of the patients. The authors observed diffuse ground-glass opacities on CT scans, which corresponded to hemosiderin-laden macrophages found in the alveolar spaces at open lung biopsy [14]. Wu et al. conducted a literature review on mutations in the SFTPC gene associated with familial interstitial lung disease, including a study of 35 patients. The primary symptoms observed were shortness of breath, dry cough, and clubbing of the fingers. The most common findings on chest CT scans included multiple intralobular reticular opacities, numerous cysts, and ground-glass opacities. The predominant histopathological pattern with open lung biopsy was usual interstitial pneumonia (UIP) [15].

Treatment for interstitial lung disease (ILD) related to SFTPC mutations includes corticosteroids, hydroxychloroquine, and other medications. These treatments can help modulate cellular mechanisms and improve the lipid composition in alveolar epithelial cells, potentially alleviating some symptoms associated with mutation. In severe cases where medical management is ineffective, lung transplantation may be considered [16,17].

## Conclusion

Surfactant protein-C deficiency is a rare lung disease characterized by a highly variable age of onset, severity of symptoms, and prognosis. The condition can manifest in different ways, ranging from respiratory distress syndrome (RDS) in neonates to interstitial lung disease (ILD) in adults. In young adults, chest CT imaging may reveal features indicative of ILD. These features can include ground-glass opacities, multiple pulmonary cysts, interlobular reticulations, and thickening of the septa. Genetic testing is an important diagnostic consideration when these findings are present, especially in patients who experience chronic respiratory symptoms or have a history of pulmonary issues that began in infancy.

## Patient consent

All patients involved in this case series provided written consent for the use of their medical information.

## References

[1] Gower W.A., Nogee L.M. (2011). Surfactant dysfunction. Paediatr Respirat Rev.

[2] Han S., Mallampalli R.K. (2015). The role of surfactant in lung disease and host defense against pulmonary infections. Ann Am Thorac Soc.

[3] Acosta-Rivera V., Melendez-Montañez J.M., Diaz-Sotomayor F., De Jesús-Rojas W. (2021). Surfactant protein C deficiency in a Puerto Rican adolescent with a rare SFTPC genetic variant. Cureus.

[4] Nogee L.M., Dunbar A.E., Wert S.E., Askin F., Hamvas A., Whitsett J.A. (2001). A mutation in the surfactant protein C gene associated with familial interstitial lung disease. N Engl J Med.

[5] Singh J., Jaffe A., Schultz A., Selvadurai H. (2021). Surfactant protein disorders in childhood interstitial lung disease. Eur J Pediatr.

[6] Mechri M., Epaud R., Emond S., Coulomb A., Jaubert F., Tarrant A. (2010). Surfactant protein C gene (SFTPC) mutation-associated lung disease: high-resolution computed tomography (HRCT) findings and its relation to histological analysis. Pediatr Pulmonol.

[7] Peca D., Boldrini R., Johannson J., Shieh J.T., Citti A., Petrini S. (2016). Clinical and ultrastructural spectrum of diffuse lung disease associated with surfactant protein C mutations. Eur J Hum Genet.

[8] Thouvenin G., Abou Taam R., Flamein F., Guillot L., Le Bourgeois M., Reix P. (2010). Characteristics of disorders associated with genetic mutations of surfactant protein C. Arch Dis Child.

[9] Cameron H.S., Somaschini M., Carrera P., Hamvas A., Whitsett J.A., Wert S.E. (2005). A common mutation in the surfactant protein C gene associated with lung disease. J Pediatr.

[10] Thomas A.Q., Lane K., Phillips J., Prince M., Markin C., Speer M. (2002). Heterozygosity for a surfactant protein C gene mutation associated with usual interstitial pneumonitis and cellular nonspecific interstitial pneumonitis in one kindred. Am J Respir Crit Care Med.

[11] Somaschini M., Presi S., Ferrari M., Vergani B., Carrera P. (2018). Surfactant proteins gene variants in premature newborn infants with severe respiratory distress syndrome. J Perinatol.

[12] Salerno T., Peca D., Rossi F.P., Menchini L., Danhaive O., Cutrera R. (2013). Bronchiectasis and severe respiratory insufficiency associated with a new surfactant protein C mutation. Acta Paediatr.

[13] Chua W.C., Chen I.C., Liu Y.C., Wu Y.H., Lo S.H., Hsu J.H. (2022). Congenital surfactant C deficiency with pulmonary hypertension-A case report. Children (Basel).

[14] Tang X., Shen Y., Zhou C., Yang H., Liu H., Li H. (2019). Surfactant protein C dysfunction with new clinical insights for diffuse alveolar hemorrhage and autoimmunity. Pediatr Investig.

[15] Wu T.T., Yu Y.M., Tang P., Zhuang Q.D., Zhang Y., Lai N.Y. (2022). [Familial interstitial lung disease associated with surfactant protein C gene mutation in adults: report of two cases and literature review]. Zhonghua Jie He He Hu Xi Za Zhi.

[16] Park J.S., Choi Y.J., Kim Y.T., Park S., Chae J.H., Park J.D. (2018). Pediatric case report on an interstitial lung disease with a novel mutation of SFTPC successfully treated with lung transplantation. J Korean Med Sci.

[17] Woischnik M., Sparr C., Kern S., Thurm T., Hector A., Hartl D. (2010). A non-BRICHOS surfactant protein c mutation disrupts epithelial cell function and intercellular signaling. BMC Cell Biol.

